# Behavioral Effects of Acute Systemic Low-Dose Clozapine in Wild-Type Rats: Implications for the Use of DREADDs in Behavioral Neuroscience

**DOI:** 10.3389/fnbeh.2018.00173

**Published:** 2018-08-14

**Authors:** Ann-Kathrin Ilg, Thomas Enkel, Dusan Bartsch, Florian Bähner

**Affiliations:** ^1^Department of Psychiatry and Psychotherapy, Central Institute of Mental Health, Medical Faculty Mannheim, Heidelberg University, Mannheim, Germany; ^2^Department of Theoretical Neuroscience, Central Institute of Mental Health, Medical Faculty Mannheim, Heidelberg University, Mannheim, Germany; ^3^Department of Molecular Biology, Central Institute of Mental Health, Medical Faculty Mannheim, Heidelberg University, Mannheim, Germany

**Keywords:** clozapine, CNO, designer receptors exclusively activated by designer drugs (DREADD), rats, locomotion, elevated plus-maze, set-shifting, social interaction

## Abstract

Designer receptors exclusively activated by designer drugs (DREADDs) are popular tools used to manipulate the activity of defined groups of neurons. Recent work has shown that DREADD effects in the brain are most likely not mediated by the proposed ligand clozapine-N-oxide (CNO) but its metabolite clozapine (CLOZ). However, it is not known whether low doses of CLOZ required to activate DREADDs already have DREADD-independent effects on behavior as described for higher CLOZ doses used in previous preclinical studies. To close this gap, we compared effects of acute systemic (i.p.) CLOZ treatment vs. vehicle (VEH) in a wide range of behavioral tests in male wild-type rats. We found that CLOZ doses as low as 0.05–0.1 mg/kg significantly affected locomotion, anxiety and cognitive flexibility but had no effect on working memory or social interaction. These results highlight the need for careful controls in future chemogenetic experiments and show that previous results in studies lacking CNO/CLOZ controls may require critical re-evaluation.

## Introduction

Designer receptors exclusively activated by designer drugs (DREADDs) are one of the most commonly used tools to manipulate activity in specific brain circuits with the goal to establish sufficiency or necessity of manipulated neurons for behavior (Roth, 2016; Smith et al., 2016). These receptors are typically activated by systemic injection of a ligand. The most commonly used DREADD is derived from the human muscarinic receptor and has been shown to bind clozapine-N-oxide (CNO), a metabolite of the antipsychotic clozapine (CLOZ), with high affinity (Armbruster et al., 2007). Furthermore, it was assumed that CNO has very low affinities to endogenous cellular substrates, penetrates the blood-brain-barrier and is not metabolized into the psychoactive substances CLOZ and N-desmethylclozapine (N-Des) in rodents (Armbruster et al., 2007), suggesting that it may be an optimal ligand for *in vivo* studies targeting the rodent brain. However, recent studies shook some of these assumptions: first, CNO is indeed back-metabolized to CLOZ and N-Des in rodents (MacLaren et al., 2016; Gomez et al., 2017). Second, in contrast to CLOZ, CNO shows extremely poor permeation of the blood-brain barrier and CLOZ appears to have a much higher affinity to DREADDs than CNO. Gomez et al. (2017) provided evidence that DREADD-specific effects seen after CNO administration are caused by binding of back-metabolized CLOZ and not the injected ligand itself. Therefore, Gomez et al. (2017) suggested the use of “subthreshold” low-dose CLOZ to activate DREADDs, thus avoiding potential side effects of high CNO doses (MacLaren et al., 2016; Gomez et al., 2017).

Indeed, the exact pharmacokinetics of systemically administered CNO is unknown and studies differ with respect to the amount of back-metabolized CLOZ measured. More specifically, MacLaren et al. (2016) reported that the plasma level of back-metabolized CLOZ was ~13% of CNO, whereas Gomez et al. (2017) measured back-metabolized CLOZ plasma levels corresponding to ~2% of CNO levels. Since the behavioral effects seen in a DREADD-experiment after CNO injection (10 mg/kg) were comparable to a 100-fold lower dose of CLOZ (0.1 mg/kg) and no side-effects on locomotor activity were observed with 0.1 mg/kg CLOZ in rats, Gomez et al. (2017) proposed the use of this CLOZ dose instead of CNO. However, a prerequisite for this approach is that the used dose of CLOZ itself does not have any DREADD-independent effects on behavior.

The antipsychotic CLOZ has various dose-dependent effects on brain physiology: it antagonizes neuronal receptors for histamine, noradrenaline, serotonin, dopamine and acetylcholine and influences local neurotransmitter concentrations, especially in the striatum and the medial prefrontal cortex (Schotte et al., 1993; Ashby and Wang, 1996). In preclinical studies, the observed effects after acute systemic application of therapeutic doses of CLOZ (i.e., 1–10 mg/kg) in rodents include decreased spontaneous locomotor activity (McOmish et al., 2012), inhibition of amphetamine-induced hypermotility (Arnt, 1995), diverse effects in tests of working memory (Hauber, 1993) as well as decreased activity in the elevated plus-maze (Cao and Rodgers, 1997). Furthermore, in rodent models of schizophrenia, acute treatment enhanced cognitive flexibility (Szlachta et al., 2017) and chronic treatment attenuated social interaction deficits (Sams-Dodd, 1996).

While CLOZ effects in rodents are well-examined for the high doses comparable to those used in human psychiatric treatment, behavioral effects caused by back-metabolized CLOZ (after CNO injections) or the proposed low-dose CLOZ administration in DREADD experiments remain largely unknown. Since most DREADD-experiments used doses between 1 mg/kg and 10 mg/kg CNO (MacLaren et al., 2016; Smith et al., 2016), plasma levels of back-metabolized CLOZ would be comparable to CLOZ injections between ~0.02 mg/kg and ~1.3 mg/kg (see above). Indeed, few studies used doses lower than 0.5 mg/kg CLOZ but these indicate that there may also be behavioral effects (Gleason and Shannon, 1997; Manzaneque et al., 2002; Szlachta et al., 2017). However, most of these studies were performed using rodent models for psychiatric disorders and the effects of low-dose CLOZ on non-impaired animals are thus unknown.

In order to close this gap, we investigated the effect of low-dose CLOZ on the behavior of male adult Sprague-Dawley rats. We performed experiments testing different behavioral domains: spontaneous locomotor activity in the open field, anxiety-related behavior using the elevated plus-maze, social interaction, working memory abilities in an operant delayed alternation task and cognitive flexibility in an operant set-shifting paradigm. All experiments were conducted with 0.1 mg/kg CLOZ since this dose has been proposed for DREADD-experiments (Gomez et al., 2017), other doses were tested depending on the outcomes. The results are relevant for the possible use of CLOZ in future experiments with DREADDs and for the evaluation of earlier studies that lacked appropriate control groups.

## Materials and Methods

### Animals

Male adult Sprague-Dawley rats (Charles River, Sulzfeld, Germany) were 4–7 months old and weighed 537 g ± 70 g (mean ± standard deviation). They were housed in standard macrolon cages (55 × 33 × 20 cm) in groups of four. We used a controlled feeding regimen (20 g per rat and day, provided after daily experiments ended) which allowed for normal weight gain but made sure that animals were motivated during experiments. Rats had free access to water. Lights were turned on from 7:30 am to 7:30 pm and experiments were performed during the light phase. For the social interaction task, 6 weeks old male partners weighing 165–210 g were used. All experiments were performed in accordance with national and international ethical guidelines, conducted in compliance with the German Animal Welfare Act and approved by the local authorities (Regierungspräsidium Karlsruhe, Germany). Efforts were made to reduce the number of animals used, and all behavioral protocols were refined to minimize adverse effects on animal well-being. Throughout the study period, no adverse health events occurred that demanded special veterinary care or removal of an animal from any experiment.

### Drugs

CLOZ (Tocris, UK) was dissolved in dimethyl sulfoxide (DMSO; Sigma-Aldrich, St. Louis, MO, USA) and diluted with 0.9% saline (SAL) to volume. Since we used minimal concentrations of DMSO to dissolve CLOZ, DMSO concentrations in the final CLOZ or vehicle/VEH solutions were 1.2% for CLOZ doses of 0.05 and 0.1 mg/kg and 1.6% for 0.3 mg/kg CLOZ/VEH. The injected volume for these doses was 0.7–1 ml/kg. A higher DMSO concentration (67%) was necessary for the dose of 1 mg/kg CLOZ (injected volume: 0.33 ml/kg). We prepared drug solutions daily and injected rats 30 min before the start of behavioral experiments.

### Behavioral Experiments

#### Locomotor Activity

The test of locomotor activity took place in an open field arena. The gray PVC arena consisted of a base divided by 50 cm high walls into four equally-sized squares (51 × 51 cm). Hence, four animals were tested simultaneously. Light intensity was 35 lx, measured at the center of the sections. Video analysis software (Viewer 2, Biobserve GmbH, Bonn, Germany) tracked the rats and calculated the distance traveled. One experimental session was performed per day. Rats (*n* = 19) were placed manually in the open field and could move freely for 30 min. After one habituation session, six sessions with intraperitoneal injections were performed. The sequence of injections was vehicle (VEH 1)—CLOZ (0.1 mg/kg)—CLOZ (1.0 mg/kg)—CLOZ (0.05 mg/kg)—VEH 2—saline (SAL) in a within-subject design. The 3rd CLOZ session (0.05 mg/kg) was performed 3 days after the 2nd session (1.0 mg/kg) to exclude residual CLOZ effects (elimination half-life from brain is estimated to be 1.5–1.6 h in rats; see Baldessarini et al., 1993). We focused on the minutes 16–30 in our analysis because we were interested in CLOZ effects on general locomotor activity as measured in the steady-state and not on initial exploratory behaviors. Moreover, such exploratory behaviors have been shown to vary across multiple experimental days (Russell and Williams, 1973). The second VEH injection served as a control for habituation effects across days and the SAL injection served as a further control because previous studies showed significant decrease of locomotor activity after injections with high DMSO concentrations in rodents (Castro et al., 1995; Markvartova et al., 2013).

#### Elevated Plus-Maze

The elevated plus-maze is an established behavioral task for testing anxiety in rodents (Pellow et al., 1985). The gray PVC maze consisted of four concentric arms (50 × 12 cm) connected by a central platform (12 × 12 cm) and was raised 50 cm above floor level. Two opposite arms surrounded by a 50 cm high wall were called “closed arms,” the other two opposing arms without walls were called “open arms.” The light intensity on the closed arms was 35 lx compared to 120 lx on the open arms. After receiving CLOZ in doses of 0.05 mg/kg (*n* = 10), 0.1 mg/kg (*n* = 9), 1 mg/kg (*n* = 10) or VEH (*n* = 13, between-subject design), rats were placed manually on the central platform, facing an open arm, and could move freely for 5 min. A video camera recorded the experiment. An entry was counted each time the rat entered an arm with all four paws. The percentage of open arm entries (%OAE) and percentage of open arm time (%OAT) was analyzed, and the total number of entries was counted to control for locomotor effects between groups (Hogg, 1996).

#### Social Interaction

The social interaction task tests motivation for social contact (Sams-Dodd, 1996). The experiment took place in one part of the open field arena described above. Rats received VEH or 0.1 mg/kg CLOZ (*n* = 10 per group, between-subject design) and the following day VEH or 0.3 mg/kg CLOZ (*n* = 10 per group, newly mixed groups). One day before injections, rats and young partner rats were separately habituated for 30 min to the arena. During the social interaction task, focal rats were placed into the arena, where they moved freely for 1 min. Then the young partner rat was placed into the arena for 5 min. Each partner rat interacted once a day and rotated between days such that all focal rats interacted with an unknown partner during both sessions. Videos of the experiment were analyzed blinded by measuring the cumulative active interaction times. Active interaction was scored as the time that rats engaged in one of the following behaviors: anogenital or non-anogenital sniffing, grooming, crawling over/under the partner and approach/following the partner (see Vanderschuren et al., 1997 for details).

#### Strategy Set-Shifting

The strategy set-shifting and delayed alternation tasks were carried out in either large (30 × 48 × 41 cm, custom-made) or standard-sized automated operant training chambers (21 × 29 × 24 cm), respectively. All procedures were controlled by a computer running MedPC-IV software and custom-made MedStat Notation code (MedAssociates Inc., Fairfax, VT, USA). The chambers (all hardware and software from Med Associates, Inc., Fairfax, VT, USA) were equipped with two retractable levers, located left and right from a central food tray, into which food rewards were delivered (set shift: 45 mg food pellets, BioServ, Flemington, NJ, USA; delayed alternation: 80 μl of sweetened condensed milk, Milchmaedchen, Nestle, Germany). Cue lights were located above each lever and a house light was placed in the upper left corner opposite the food tray. All chambers were light- and sound-attenuated and a ventilator provided constant background noise.

The strategy set-shifting paradigm is a test for cognitive flexibility and was performed by adapting procedures described in Floresco et al. (2008). Initially, rats (*n* = 14) were trained to respond equally to the presentation of both levers individually. During the actual task, rats first learned to respond according to a visual rule (i.e., “press lever with illuminated cue light above”) on two consecutive days with 100 trials each. On the next 3 days, rats first performed the visual rule (“baseline”), followed by two unsignaled rule switches: first to a place rule (“always press the lever on one side, ignore cue light,” i.e., a cue → place shift) and then back to using the visual rule again (place → cue shift). Rules switched after a rat had reached a performance criterion (18/20 trials correct). Rats were then divided into two groups (*n* = 7 per group), matched according to their performance during the 3rd cue → place shift. On the next day, rats received either 0.1 mg/kg CLOZ or VEH (between-subject design) and performed the set-shift as on previous days. Performance in both groups was compared using the trial numbers required to reach criterion. Furthermore, types of errors were analyzed as described in Floresco et al. (2008). Briefly, errors were subdivided into old errors and never-reinforced errors. Old errors are incorrect decisions that would be correct according to the previous rule. These were further subdivided into perseverative (number of old errors at the beginning of the new rule) and regressive errors (number of old errors after a certain performance level has already been reached; this is a measure for the ability to maintain a new strategy; see Floresco et al., 2008 for details). Errors were classified as never-reinforced if the response was neither compatible with the current nor the previous rule.

#### Delayed Alternation Task

The delayed alternation task was adapted from procedures described by Dunnett et al. (1999). Initially, rats (*n* = 19) were trained to press the left and right levers in separate sessions. During “alternation training,” each trial consisted of the presentation of both levers; however, lever extension was triggered by nose poking into the food tray to center rats between the levers to avoid the use of a place strategy. Rats had to learn to alternate between responding on the left and right lever, e.g., when a rat had responded on the left lever and received a reward, on the next trial a response on the right lever was required for another reward (correct alternation); repeating a lever press on the same side was defined as an incorrect response and no reward was delivered. After making an incorrect response, rats had to press on the correct side before proceeding with the task. Alternation training consisted of 80 trials/day (or a maximum of 30 min) and was continued until stable alternation behavior had been established (>80% correct on two consecutive days; mean: 9 ± 3 days). In the final “delayed alternation” phase, delays (5–25 s) were randomly inserted between successive lever presentations (80 trials or max. 40 min/session). Rats were then tested over 4 days in the delayed alternation procedure, receiving either VEH or CLOZ (0.1 mg/kg) for 2 days in a crossover within-subject design. Performance was analyzed as the percentage of correct alternations across a range of actually experienced (i.e., not programed) delays.

### Data Analysis

Data analysis was performed with Graphpad Prism (Version 7), Microsoft Excel (2010) and Matlab (R2017a). Parametric tests were used if groups passed a normality test, and otherwise, non-parametric statistics was used. The Mann-Whitney U test was used to compare the performance of CLOZ and VEH groups in the strategy set-shifting and social interaction task. Data from the elevated plus-maze was analyzed using the non-parametric Kruskal-Wallis test and Dunn’s test for multiple comparisons was employed for pairwise *post hoc* tests. Data from the open field task was analyzed using one-way repeated-measures ANOVA, followed by Dunnett’s test for multiple comparisons to compare all treatments with the first VEH treatment. Performance in the delayed alternation task was analyzed using repeated-measures ANOVA with within-subject factors treatment (VEH, CLOZ) and delay (10–15 s, 15–20 s, 20–25 s, 25–30 s and 30–35 s). The significance threshold was set at *p* < 0.05. Data collected in the open field and delayed alternation task are displayed as mean ± standard error of the mean/SEM. All other data were presented as median and first and third quartile (P_25_, P_75),_ Tukey-style whiskers were used in boxplots. Outliers are defined as values >P_75_ + 1.5 × interquartile range/IQR and <P_25_ − 1.5 × IQR, respectively, and shown as individual dots.

## Results

### Clozapine Reduces Locomotor Activity and Induces an Anxious Phenotype but Does Not Affect Social Interaction

**Figure 1:**
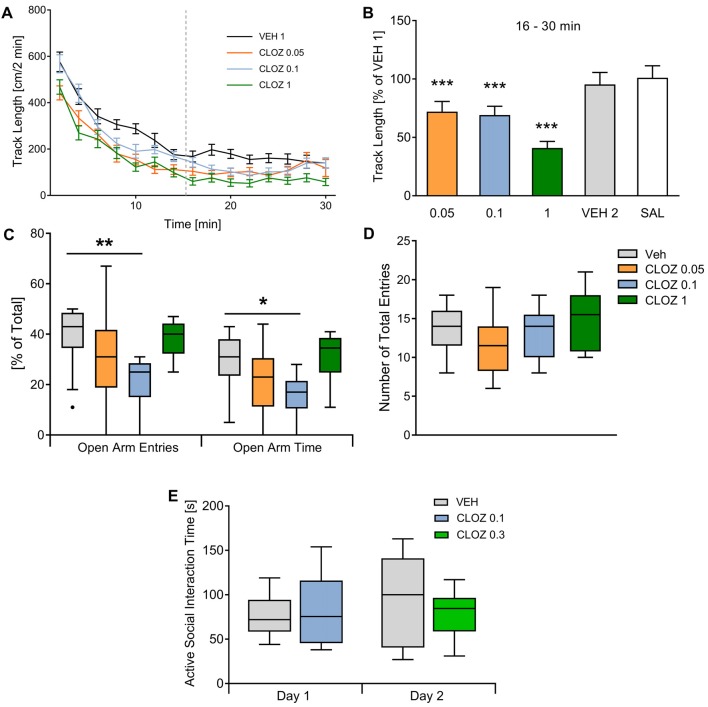
Clozapine/CLOZ reduces locomotor activity and induces an anxious phenotype but does not affect social interaction. **(A)** Locomotor activity: directly after placing rats (*n* = 19) in the open field, they showed high locomotor activity and reached a steady state after 15 min (data of the second vehicle injection/VEH 2 and saline/SAL were not different from VEH and are not shown to improve readability of the figure). **(B)** Track length in all conditions is expressed as percentage of the VEH 1 baseline value. For this steady-state activity, track length was affected by subject (repeated-measures one-way ANOVA followed by Dunnett’s test; *F*_(18,90)_ = 8.85; *p* = 3 × 10^−13^) and treatment (*F*_(5,90)_ = 16.34; *p* = 2 × 10^−11^). We observed a dose-dependent decrease after low-dose CLOZ administration (all compared against VEH injection). The lowest doses used caused similar decreases in track length (0.1 mg/kg CLOZ: *p* = 2 × 10^−4^, this corresponds to 69.2 ± 7.5% of the track length after VEH injection; 0.05 mg/kg CLOZ: *p* = 10^−4^; 72.1 ± 8.73%). There was an even stronger reduction with 1 mg/kg CLOZ (*p* < 10^−4^; 40.9 ± 5.7%). The second vehicle (VEH 2) injection was performed to test if repetition influences steady-state activity, but no difference was observed (*p* = 0.27; 95.4 ± 10.2%). Also, SAL treatment didn’t change the track length (*p* = 0.56; 101.1 ± 10.2%). Data are represented as mean ± standard error of the mean (SEM). **(C,D)** Elevated plus-maze: the percentage of open arm entries/%OAE (*H* = 13.0, *p* = 0.0046, Kruskal-Wallis test) and the percentage of open arm time/%OAT (*H* = 11.3, *p* = 0.010, Kruskal-Wallis test) was different between groups. *Post hoc* tests showed that the dose of 0.1 mg/kg induced an anxious phenotype. Both the %OAE (*p* = 0.023, Dunn’s multiple comparisons test) and %OAT (*p* = 0.023) were decreased with this dose (*n* = 9). 0.05 mg/kg and 1 mg/kg CLOZ did not affect the %OAE or the %OAT significantly. The total number of entries (controlling for locomotor activity effects) were not different between groups (*p* = 0.24, Kruskal-Wallis test). **(E)** Social interaction: no significant differences concerning cumulative active social interaction time were observed with both doses tested (0.1 mg/kg and 0.3 mg/kg CLOZ, *p* = 0.99 and *p* = 0.57, *n* = 10 per group). Data displayed as median and the first and third quartile (P_25_, P_75_; Tukey-style whiskers) *p < 0.05, **p < 0.01, ***p < 0.001.

We compared the effects of low-dose CLOZ vs. VEH on male adult Sprague-Dawley rats in several behavioral domains including locomotor activity, anxiety, social interaction, set-shifting and working memory. We first investigated the effect of CLOZ on locomotor activity in the open field using a within-subject design (*n* = 19; Figures 1A,B). Rats placed in the open field showed high activity until reaching a steady-state. Analysis showed that steady-state activity (16–30 min) was affected by subject (*F*_(18,90)_ = 8.85, *p* = 3 × 10^−13^) and treatment (*F*_(5,90)_ = 16.34, *p* = 2 × 10^−11^, repeated-measures one-way ANOVA). Steady-state track length decreased after CLOZ administration in a dose-dependent fashion. At a dose of 0.1 mg/kg, the track length significantly decreased when compared to VEH (*p* = 2 × 10^−4^, 69.2 ± 7.5% of baseline value). A similar decrease was observed with 0.05 mg/kg (*p* = 10^−4^, 72.1 ± 8.7% VEH 1 baseline value). As expected, a dose of 1 mg/kg CLOZ decreased the track length even further (*p* < 10^−4^, 40.9 ± 5.7% of baseline). There were no differences between the first and second VEH session (*p* = 0.27, 95.4 ± 10.2% of baseline), confirming that the observed CLOZ effects were not confounded by between-days habituation.

Next, we examined whether CLOZ influences anxiety as measured in the elevated plus-maze using the same doses as above in a between-subject design (Figures 1C,D). The percentage of open arm entries (%OAE) differed between groups (*H* = 13.0, *p* = 0.0046, Kruskal-Wallis test, Figure 1C). Compared to VEH (*n* = 13), just the dose of 0.1 mg/kg CLOZ induced an anxious phenotype (*p* = 0.0023, Dunn’s multiple comparison test used for pairwise *post hoc* tests, *n* = 9), whereas the lowest dose (0.05 mg/kg CLOZ, *p* = 0.25, *n* = 10) and the highest dose (1 mg/kg CLOZ, *p* > 0.99, *n* = 10) didn’t affect the %OAE significantly. Similarly, the percentage of open arm time (%OAT) differed significantly between groups (*H* = 11.3, *p* = 0.010, Kruskal-Wallis test, Figure 1C). Just as %OAE, the %OAT was decreased with the dose of 0.1 mg/kg CLOZ (*p* = 0.023) but was not affected at other doses (0.05 mg/kg CLOZ: *p* = 0.47; 1 mg/kg CLOZ: *p* > 0.99). There was a trend towards increased anxiety-related behavior in the 0.05 mg/kg group as compared to VEH for both %OAE (*p* = 0.08) and %OAT (*p* = 0.16) if pairwise *post hoc* tests were not controlled for multiple comparisons. The total entries were not different between groups (*H* = 4.2, *p* = 0.24, Kruskal-Wallis test, Figure 1D).

The use of DREADDs is also especially interesting for examining the neurobiological basis of unrestrained social behaviors. We therefore examined whether CLOZ alone influences social interaction with a younger same-sex partner in the open field (Figure 1E). Both CLOZ doses tested did not affect active social interaction time when compared to VEH (0.1 mg/kg and 0.3 mg/kg CLOZ; *n* = 10 per group, between-subject design, *p* = 0.99 and *p* = 0.57, Mann-Whitney U test).

### Clozapine Increases Cognitive Flexibility but Does Not Affect Working Memory Performance

**Figure 2:**
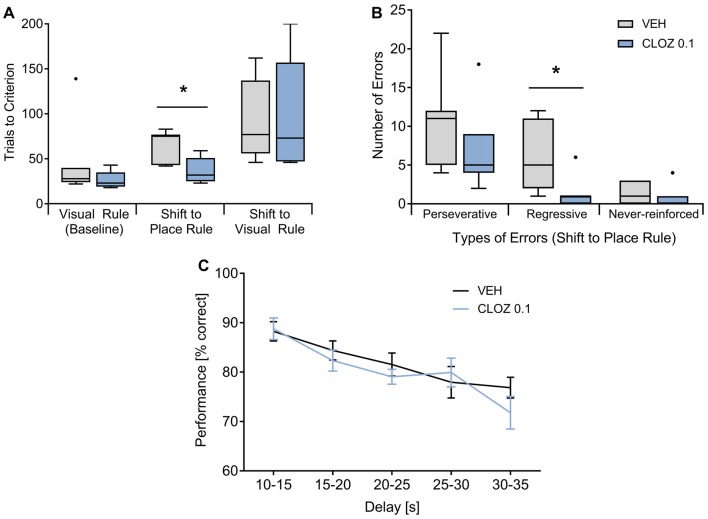
CLOZ increases cognitive flexibility but does not affect working memory performance. **(A,B)** Strategy set-shifting: during the visual baseline, rats (*n* = 7 per group) did not differ with respect to performance, whereas they reached the criterion during the shift from the visual to the place rule in fewer trials (CLOZ 0.1 mg/kg vs. VEH; *p* = 0.011). Trials to criterion were not different for the shift back to visual rule. The better performance during the shift to the place rule went along with a lower number of old errors (i.e., responses that are correct according the previous rule; *p* = 0.016). This was caused by a decrease in the number of regressive (*p* = 0.016) but not perseverative errors, indicating that CLOZ improves rule maintenance after the switch. The number of never-reinforced errors was not different. Data shown as median (P_25_, P_75_). *p < 0.05. **(C)** Delayed alternation task: performance decreased significantly when longer delays separated two consecutive choice trials (*F*_(4,72)_ = 9.42; *p* = 4 × 10^−6^), but there was no difference between VEH or CLOZ treatment (*F*_(1,18)_ = 0.93; *p* = 0.347; *n* = 19 rats). Data are expressed as mean ± SEM.

We also assessed whether low-dose CLOZ alters executive functions as measured in operant chambers using both a strategy set-shifting task (Floresco et al., 2008) and a delayed alternation working memory paradigm (Dunnett et al., 1999). In the two-choice set-shifting task, rats were treated with either 0.1 mg/kg CLOZ or VEH (*n* = 7 per group, between-subject design; Figures 2A,B). During the visual rule baseline, performance did not differ between groups. However, rats treated with CLOZ reached the performance criterion during the 1st shift (visual cue → place rule) in fewer trials (*p* = 0.011, Mann-Whitney U test). Trials to criterion during the 2nd shift (place → cue rule) were not different. This indicates that the rule switching phenotype in the CLOZ group indeed reflects increased cognitive flexibility and was not caused by baseline differences. The better performance during the shift to the place rule went along with a lower number of errors of the regressive type (*p* = 0.016), indicating that CLOZ improves performance by facilitating the maintenance of the place rule after the first rule switch (Floresco et al., 2008). The number of perseverative or never-reinforced errors was not different.

In the delayed alternation task, the analysis (repeated-measures ANOVA treatment × delay) was based on a total of 135 ± 7.8 or 125 ± 10.6 trials per rat for VEH or 0.1 mg/kg CLOZ, respectively (*n* = 19, within subject design; Figure 2C). Performance was significantly affected by the factor delay (*F*_(4,72)_ = 9.42; *p* = 4 × 10^−6^), suggesting dependency on working memory processes, but was not different under VEH or CLOZ treatment (*F*_(1,18)_ = 0.93; *p* = 0.347). Further, there was no interaction of these factors (*F*_(4,72)_ = 1.25; *p* = 0.297). The same dose of CLOZ (0.1 mg/kg) thus improved cognitive flexibility in the set-shifting task but did not alter working memory performance.

In summary, acute CLOZ administration at doses recommended for DREADD activation and 10–100× lower than those previously used in preclinical studies already has unexpected but significant effects in several behavioral domains.

## Discussion

Previous behavioral studies using CLOZ have focused on doses comparable to the therapeutic range used in humans (Baldessarini et al., 1993). In contrast, low-dose CLOZ effects (i.e., <0.5 mg/kg) have received little attention and it is unclear whether such doses already have DREADD-independent effects. However, this is an important issue because a recent publication (Gomez et al., 2017) that received widespread attention suggested that not CNO but its metabolite CLOZ mediates DREADD-specific effects. These and other findings (including potential DREADD-independent effects of CNO; MacLaren et al., 2016; Gomez et al., 2017) led to the conclusion that low-dose CLOZ should instead be used as a ligand (but see: Mahler and Aston-Jones, 2018). Furthermore, many previous studies using DREADDs haven’t included a CNO/CLOZ control group but rather used a within-subject design (discussed in MacLaren et al., 2016). We therefore investigated the effects of acute CLOZ treatment in male wild-type Sprague-Dawley rats and indeed found reduced locomotor activity in the open field, increased anxiety-related behavior in the elevated plus-maze task and improved cognitive flexibility during the strategy set-shifting task at doses used in DREADD experiments (Gomez et al., 2017). Working memory performance in the delayed alternation task and spontaneous social interaction were not affected.

Since CLOZ acts on multiple receptors in many different brain areas, behavioral effects are unlikely to map onto a single brain region or neurotransmitter system. However, CLOZ binds to some receptors with higher affinity than to others as reflected by lower ED_50_-values. Low ED_50_-values have been reported for H_1_ (0.15 mg/kg), α_1_ (0.58 mg/kg), 5-HT_2_ (depending on study 1.3 mg/kg or 0.19 mg/kg) and 5-HT_1C_ receptors (1.8 mg/kg), whereas higher ED_50_-values are observed for D_2_, α_2_, mACh and 5-HT_1A_ receptors (ED_50_ > 9 mg/kg; Schotte et al., 1993; Natesan et al., 2007). Thus, behavioral effects of CLOZ should also vary in a dose-dependent fashion. More specifically, it is possible that low-dose CLOZ effects are only observed in paradigms that are sensitive to antagonism of receptors that CLOZ binds with high affinity.

Our complex pattern of results matches those from previous studies using either specific 5-HT_2A_R antagonists or CLOZ in 5-HT_2A_R knock-out mice. First, it was shown that the suppression of locomotor activity induced by CLOZ is mediated by a 5-HT_2A_R mechanism (McOmish et al., 2012). Second, 5-HT_2A_R blockade increases anxiety-related behavior in the rat elevated plus-maze (Setem et al., 1999). Third, 5-HT_2A_R blockade facilitates the shift from visual to place rule in rats (Baker et al., 2011). Last, neither social interaction nor working memory abilities of rats were affected by selective 5-HT_2A_R blockade (Kennett, 1992; Costall and Naylor, 1995; Ruotsalainen et al., 1997). Thus, the antagonism of 5-HT_2A_R after CLOZ administration could account for the observed pattern of results, but as CLOZ binds to many receptors this remains a speculative hypothesis and will be discussed in detail below. Table 1 provides a summary of behavioral findings after antagonism of 5-HT_2A_, H_1_ and α_1_ receptors in paradigms that are similar to the ones employed in this study. These receptors were chosen because their ED_50_ values for CLOZ (<1 mg/kg) match the CLOZ dose range we used in this study.

**Table 1:** Effects of selective antagonists for receptors that Clozapine/CLOZ binds with highest affinity (ED_50_ <1 mg/kg) on behaviors tested in this study.

Receptor antagonism*	Locomotor activity	Elevated plus-maze	Social interaction	Cognitive flexibility	Working memory
H_1_ receptor	**Decrease** (intraventricular injection in fowls; Nisticò et al., 1980) **Decrease** (24 h measurement of circadian rhythm in H1R-knock-out mice; Inoue et al., 1996)	**Anxiogenic** (mice; Serafim et al., 2013) **Anxiolytic** (attenuation of L-histidine-induced anxiety in mice; Kumar et al., 2007)	**No effect** (rats; Kennett, 1992)		**Impairment** (eight-arm radial maze spatial memory test in rats; Taga et al., 2001) **Impairment** (intrahippocampal injection before eight-arm radial maze delayed spatial win-shift task in rats; Okada et al., 2010)
5-HT_2A_ receptor	**Decrease** (inhibition of PCP-induced hyperlocomotion in rats; Krebs-Thomson et al., 1998) **Mediation of CLOZ-induced locomotor suppression** (in knock-out mice; McOmish et al., 2012)	**Anxiogenic** (rats; Setem et al., 1999)	**No effect** (rats; Kennett, 1992; Costall and Naylor, 1995)	**Improvement of shift from visual- to place rule** (cross-maze version of attentional set-shift in rats; Baker et al., 2011)	**No effect** (operant delayed non-match to position task in rats; Ruotsalainen et al., 1997)
α_1_ receptor	**Decrease** (inhibition of amphetamine-induced hyperlocomotion in mice; Snoddy and Tessel, 1985)	**Anxiolytic** (inhibition of alcohol-induced anxiety in rats upon chronic administration; Skelly and Weiner, 2014) **Anxiolytic** (Inhibition of alcohol deprivation-induced anxiety in rats; Rasmussen et al., 2017)		**No effect** (cross-maze version of attentional set-shift in rats; Baker et al., 2011)	**No effect** (operant delayed non-match to position task in rats; Puumala and Sirviö, 1997)

*Unless otherwise stated, administration of selective antagonists was acute and systemic.

Our finding of a dose-dependent decrease of locomotion in the open field is consistent with previous literature given that CLOZ effects on locomotion are abolished in 5-HT_2A_ receptor knock-out mice (McOmish et al., 2012) and the CLOZ dose range used in the current study (0.05–1.0 mg/kg) is expected to result in an increasing 5-HT_2A_ receptor blockade (systemic administration of 0.1 mg/kg CLOZ in rats leads to a prefrontal 5-HT_2_R occupancy of ~35% (Natesan et al., 2007)). Moreover, H_1_ and α_1_ receptor blockade also have been reported to decrease locomotion (see Table 1, Nisticò et al., 1980; Snoddy and Tessel, 1985; Inoue et al., 1996). An earlier study in mice indeed reported that 0.3 mg/kg CLOZ reduces phencyclidine-induced hyperlocomotion (Gleason and Shannon, 1997). In contrast to our results, Gomez et al. (2017) found no DREADD-independent effects of 0.1 mg/kg CLOZ on locomotor activity in rats. This may be explained by differences in the experimental procedures used in both studies. More specifically, while Gomez et al. (2017) placed the rats into locomotor activity chambers for 30 min directly after injection, we waited 30 min before placing them into the open field. Since peak CLOZ-concentration in the brain is measured 30 min after i.p. injection (Baldessarini et al., 1993), the maximum locomotor effect may have occurred after Gomez et al. (2017) ended their observations. Moreover, our findings are unlikely to be an artifact because locomotor effects were clearly dose-dependent and an additional VEH session excluded confounding order effects.

The increased anxiety-related behavior in the elevated plus-maze and the improved cognitive flexibility in the strategy set-shifting task after low-dose CLOZ administration were unexpected. CLOZ effects on anxiety-related behaviors in the elevated plus-maze are complex and it is still controversial whether acute CLOZ has anxiolytic properties as previously suggested (see e.g., “Discussion” section in Mead et al., 2008) Support for our findings comes from earlier studies that evaluated acute CLOZ effects in the elevated plus-maze in mice and found anxiogenic effects with low doses (0.2 mg/kg; Manzaneque et al., 2002) and effects on locomotion but not anxiety with higher doses (0.3–6 mg/kg; Cao and Rodgers, 1997).

We found a U-shaped dose-response relationship between CLOZ and anxiety. One possible explanation for this comes from the fact that 5-HT_2A_ (see above) and H_1_ receptor blockade has anxiogenic effects in the elevated plus-maze (Serafim et al., 2013) while α_1_ receptor antagonism is rather anxiolytic in this test (Komaki et al., 2014; Skelly and Weiner, 2014; Rasmussen et al., 2017). The observed net effect on anxiety could thus be a function of the differential contribution of these receptor types depending on CLOZ binding. We don’t believe that our findings are confounded by locomotor effects given that the total number of arm entries, an indicator of locomotor activity (Hogg, 1996), was not different between groups (see Figure 1D). Further, if anxiogenic effects would be better explained by decreased locomotion, then they should also increase with dose (given our results from the open field test).

CLOZ effects on cognitive flexibility have been studied in the context of rodent models for psychiatric disorders. Studies with chronic and subchronic administration between 2.5 mg/kg and 10 mg/kg CLOZ in rat models of schizophrenia showed improvements in reversal learning and the set-shifting task (Li et al., 2007; McLean et al., 2008). A recent study in mice with ketamine-induced deficits showed improvements in the set-shifting task with a low-dose of 0.3 mg/kg CLOZ (acute and subchronic) but impairments were observed after a dose of 1 mg/kg CLOZ (Szlachta et al., 2017). Therefore, the net behavioral effects again likely depend on dose-dependent binding of CLOZ at receptors with different affinities. Similar to our results, a study showed that 5-HT_2A_R blockade (but not blockade of either α_1_- or 5-HT_2C_-receptors) in healthy rats performing a cross-maze version of the set-shifting task did not influence visual rule baseline performance but facilitated a shift to the place rule which went along with a specific decrease of regressive errors (Baker et al., 2011). This study also found improvements during a shift to the visual rule which we did not observe. In line with previous observations (Floresco et al., 2008), we suggest that this difference may be explained by the high variability of performance observed in the operant version of this particular rule switch (see Figure 2A). This indicates that for a shift to the visual rule, the maze-based procedure may be more sensitive to detect pharmacological effects (Floresco et al., 2008). It is currently unknown how 5-HT_2A_Rs affect cognitive flexibility but these receptors are abundantly expressed in brain regions that are important for set-shifting like medial prefrontal cortex or striatum (Barnes and Sharp, 1999; Floresco et al., 2009) and 5-HT_2A_Rs in the medial prefrontal cortex have been shown to affect executive processing (e.g., response inhibition, see Winstanley et al., 2003).

In summary, our behavioral results using acute CLOZ injections stress the importance of including a CLOZ control group using the smallest dose that induces DREADD-specific effects in each specific experimental condition. In the future, it would also be interesting to investigate whether these findings extend to the case of chronic treatment with a DREADD ligand (see e.g., Donato et al., 2017 for a recent example using this approach in a developmental study). As it is controversial whether the continued use of acute CNO would be a better alternative (MacLaren et al., 2016; Gomez et al., 2017; Mahler and Aston-Jones, 2018), the development of new ligands that are pharmacologically inert is needed. Indeed, a recent *in vitro* study (Chen et al., 2015) found that two compounds (compound 21 and perlapine) may be useful in that respect. Future work will have to show whether these ligands are suitable for *in vivo* studies. In addition, a novel DREADD derived from an opioid receptor along with the specific ligand salvinorin B (Vardy et al., 2015) already has shown promising results. However, salvinorin B is also a weak agonist at wild-type opioid receptors (Vardy et al., 2015) and it is therefore mandatory to include appropriate controls as well to exclude off-target effects.

## Data Availability

The raw data supporting the conclusions of this manuscript will be made available by the authors, without undue reservation, to any qualified researcher.

## Author Contributions

A-KI, TE and FB designed the study; analyzed the data. A-KI and TE performed the experiments. A-KI and FB wrote the first draft. TE and DB wrote sections of the manuscript. All authors contributed to manuscript revision, reading and approved the submitted version.

## Conflict of Interest Statement

The authors declare that the research was conducted in the absence of any commercial or financial relationships that could be construed as a potential conflict of interest.
